# Orthostatic Hypotension and Cognitive Function in Individuals 85 Years of Age: A Longitudinal Cohort Study in Sweden

**DOI:** 10.14336/AD.2024.0205

**Published:** 2024-02-05

**Authors:** Peder af Geijerstam, Katie Harris, Maria M. Johansson, John Chalmers, Katarina Nägga, Karin Rådholm

**Affiliations:** ^1^Department of Health, Medicine and Caring Sciences, Faculty of Medicine and Health Sciences, Linköping University, Linköping, Sweden.; ^2^The George Institute for Global Health, University of New South Wales, Sydney, Australia.; ^3^Department of Activity and Health, and Department of Health, Medicine and Caring Sciences, Linköping University, Linköping, Sweden.; ^4^Department of Acute Internal Medicine and Geriatrics, and Department of Health, Medicine and Caring Sciences, Linköping University, Linköping, Sweden.

**Keywords:** Orthostatic hypotension, mortality, cognitive decline

## Abstract

Orthostatic hypotension (OH) is more common in the elderly and associated with increased mortality. However, its implications for 85-year-olds are not known. In the prospective observational cohort study Elderly in Linköping Screening Assessment (ELSA 85), 496 individuals in Linköping, Sweden, were followed from age 85 years with cognitive assessments. Blood pressure (BP) was measured supine and after 1, 3, 5, and 10 minutes of standing. Participants with a BP fall of ≥20 mmHg systolic or ≥10 mmHg diastolic after 1 or 3 minutes were classified as classical continuous or classical transient OH depending on whether the BP fall was sustained or not, at subsequent measurements. Those with a BP fall of the same magnitude, but only after 5 or 10 minutes were classified as delayed OH. Of participants, 329 took part in BP measurements and were included. Of these, 156 (47.4%) had classical OH (113 [34.3%] continuous classical, 38 [11.6%] transient classical), and 15 (4.6%) had delayed OH. Cognitive assessments were not markedly different between groups. After 8.6 years, 195 (59.3%) of the participants had died, and delayed vs no OH was associated with twice the risk of all-cause mortality, HR 2.15 (95% CI 1.12-4.12). Transient classical OH was associated with reduced mortality, HR 0.58 (95% CI 0.33-0.99), but not after multiple adjustments, and continuous classical OH was not associated with mortality. OH may have different implications for morbidity and mortality in 85-year-olds compared with younger populations.

## INTRODUCTION

Orthostatic hypotension (OH) is a condition with a reduction in blood pressure (BP) upon standing that is more common in the elderly, affecting up to 30% of those 75 years or older [1]. It is also associated with frailty, polypharmacy, an increased risk of falls and fractures, as well as cardiovascular events [1-5]. In previous studies of mainly individuals in their 70’s or younger, both classical and delayed OH were also associated with increased mortality [3-5].

In the next three decades, the number of individuals aged above 80 years is predicted to grow to almost half a billion people and better knowledge of their morbidities is necessary to achieve adequate care [6].

OH is defined as a reduction in BP of at least 20 mmHg systolic or at least 10 mmHg diastolic after adopting a standing position, and it can be categorized as “classical” or “delayed” according to whether the BP drop occurs before or after 3 minutes of standing upright, respectively [1-3, 7]. If the concomitant heart rate reaction is blunted, orthostatic hypotension can also be categorized as neurogenic, which is associated with an increased risk of neurodegenerative disease, including Parkinson’s disease and dementia with Lewy bodies, and which correlates with the severity of cognitive deficits [1, 2].

We aimed to assess the prevalence of classical and delayed OH in 85-year-olds in Linköping, Sweden, and its association with all-cause mortality and changes in cognitive function over time.

## MATERIALS AND METHODS

### Study population

In the prospective, observational cohort study - Elderly in Linköping Screening Assessment (ELSA 85), all 650 individuals born in 1922 in the municipality of Linköping, Sweden, were invited to participate 2 months after their 85^th^ birthday, from March 2007 to March 2008. Of these, 496 individuals accepted participation and were assessed at 85, 86, 90, and 93 years of age using questionnaires, as well as anthropometric measurements, clinical physiology, and blood analyses, Supplementary Figure 1. Office visits took place at the geriatric outpatient clinic in Linköping. In the current study, all participants for which at least one standing BP measurement was obtained were included. Baseline measurements of body mass index (BMI) and blood samples, as well as assessment of atrial fibrillation, are described in the supplementary material.

### Blood pressure and pulse measurements

Office BP and pulse were measured manually in the supine position after 5 minutes’ rest. The participants were then asked to adopt the standing position, and measurements were repeated after 1, 3, 5, and 10 minutes of the participant standing upright.

Analysis of measurements is described in detail in the supplementary material. In brief, participants were categorized as having classical OH, delayed OH, or no OH based on the BP measurements. Classical OH was defined as a decrease in BP of at least 20 mmHg systolic or at least 10 mmHg diastolic within the first 3 minutes of standing up; delayed OH was defined as not classical OH and a decrease in BP of at least 20 mmHg systolic or at least 10 mmHg diastolic within 5 to 10 minutes of standing up. Participants with neither classical nor delayed OH were categorized as “no OH”. Classical OH was further categorized as continuous or transient depending on whether the measurements at 5 and/or 10 minutes continued to fulfill the criteria of OH or not, respectively.

During the office visit, participants were asked whether they have any symptoms of OH (such as dizziness, lightheadedness, and fainting) as well.

### Assessments of fall risk and functional ability

The assessments are described in detail in the supplementary material. In brief, the Downton Fall Risk Index (DFRI) was presented both as a continuous variable and as a dichotomous variable with a cut-off at or above 3 points to indicate an increased fall risk [8]. The Timed Up and Go test score was presented both as a continuous variable and as a dichotomous variable with a cut-off of 13.5 seconds or more to indicate an increased fall risk [9]. Both the EQ-5D, the Instrumental Activity Measure (IAM), and the Personal Activities of Daily Living (PADL) questionnaire responses were reported as manages vs does not manage all items/activities independently.

### Assessments of cognitive function

The Mini-Mental State Examination (MMSE) was reported as a continuous variable, as the cut-off varies significantly between studies and populations [10]. The Cognitive Assessment Battery is a set of cognitive tests that covers the domains of speed and attention, learning and episodic memory, visuospatial and executive functions, and language, which has previously been described in detail [11]. It comprises of the New York University Paragraph Recall Test (NYU-PRT), the Symbol Digit Modalities Test, the 30-item Naming Test, a simplified version of the Token Test, a combination of the Clock Drawing Task (CLOX) Test and the Cube Copying Test, the Trail Making Test A, the Rey-Complex Figure Test, the Victoria Stroop Test, and the Parallel Serial Mental Operations (PaSMO) Test. All tests were reported in their entirety, except for the Symbol Digit Modalities Test, whereby only the score for the number of correct items was used. The change in assessments of cognitive function between baseline and follow-up after 5 years were calculated by subtracting the baseline score from the score at follow-up.

### Statistical analyses

Baseline characteristics were summarized by OH status: classical (all, transient, and continuous), delayed, or no OH. Continuous variables approximately normally distributed by visual assessment were shown as the mean and standard deviation, and differences between groups were tested using a two-sample *t*-test. Continuous variables with skewed distributions were shown as the median and interquartile interval, and differences between groups were tested using a Mann-Whitney U test. Categorical variables were shown as the frequency and percentage, and differences between groups were tested using a Chi-squared test. Blood pressure measurements and pulse in both the supine and standing positions were presented as mean and 95% CI by OH status at baseline.

Cross-sectionally, the association of baseline OH status for participants living in a nursing home, having neurological disease or dementia, atrial fibrillation, elevated supine BP, abnormal pulse response, symptoms of OH, using medications, and scoring above cut-off for fall risk assessment scores were analyzed using multinomial logistic regression and presented as odds ratios (OR) and 95% CI. Furthermore, the association of baseline OH status with systolic and diastolic supine BP, number of health care contacts and assessment scores for fall risk, functional ability, and cognitive functions were analyzed using linear regression and presented as mean and 95% CI. All analyses were crude, adjusted for sex (model 2), and adjusted for sex, BMI, living in a nursing home, atrial fibrillation, comorbidities (hypertension, diabetes mellitus, heart failure, previous cardiovascular events, and neurological disease or dementia), systolic and diastolic supine BP, polypharmacy, use of sedatives, and use of antidepressants (model 3).

The association of baseline OH status with changes in assessments of cognitive functions from baseline to follow-up were tested using linear regression with the same adjustments as above.

Cox proportional hazard models, with the same adjustments as above, were used to model the association between OH status at baseline and the risk of all-cause mortality.

**Figure 1.**
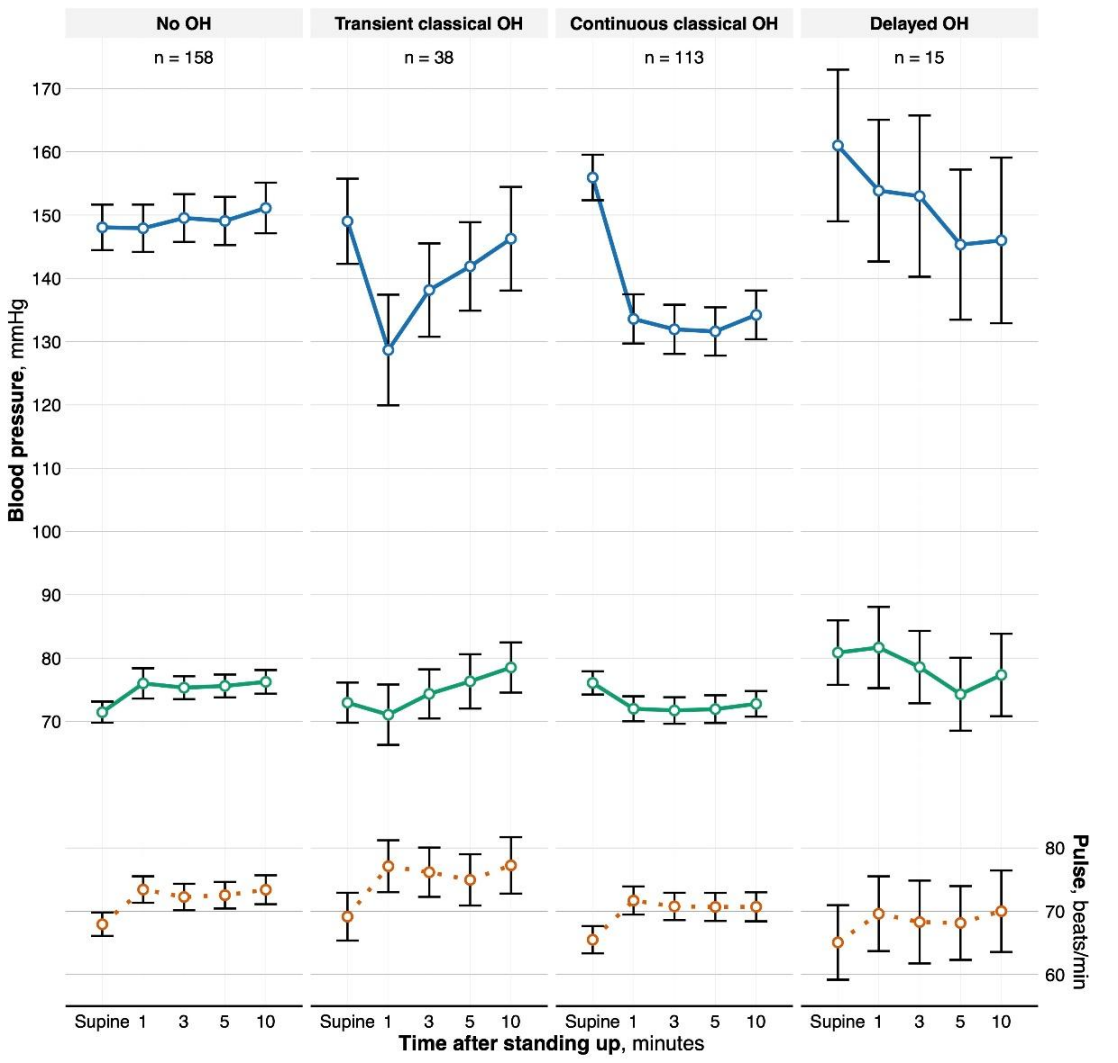
**Baseline systolic (upper solid, blue line) and diastolic (lower solid, green line) blood pressure (left y axis) as well as pulse (dotted orange line, right y axis) in the supine position and after 1, 3, 5, and 10 minutes of standing up, respectively, for each of the orthostatic hypotension classifications**. The plot includes points representing the mean values and whiskers extending between the upper and lower 95% CI. OH, orthostatic hypotension.

Statistical tests were two-tailed and *P* values of <.05 were considered statistically significant. R version 4.3.1 (R Core Team, Vienna, Austria) and RStudio version 2023.09.0+463 (Posit Software, Boston, MA, USA) were used for data analyses.

### Ethical considerations

The ELSA 85 study was approved by the Research Ethics Committee of Linköping University, Sweden (Dnr 141-06) and adheres to the Declaration of Helsinki. All participants gave written informed consent.

## RESULTS

Of 496 participants, 146 (29.4%) declined further participation, 12 (2.4%) died before the office visit, and 9 (1.8%) did not participate in any of the orthostatic BP measurements. Thus, 329 participants were included in our analysis, Supplementary Figure 1. The prevalence of classical and delayed OH was 156 (47.4%) and 15 (4.6%), respectively, and the remaining 158 (48.0%) had normal orthostatic BP. Of those with classical OH, 38 (24.4%) had transient, 113 (72.4%) had continuous, and 5 (3.2%) could not be classified further as measurements from both 5 and 10 minutes were missing, Table 1, Figure 1, and Supplementary Table 1.

**Table 1:** Baseline characteristics in relation to orthostatic hypotension status.

	All participants, N = 329	No OH, n = 158	Classical OH,			Delayed OH, n = 15
			All^1^, n = 156	Transient, n = 38	Continuous, n = 113	
**Sex (women), n (%)**	191 (58.1)	99 (62.7)	87 (55.8)	23 (60.5)	59 (52.2)	5 (33.3)
**Body mass index (kg/m^2^), mean (SD)**	26.0 (23.0-28.0)	26.0 (23.2-28.0)	25.8 (23.0-28.0)	25.0 (23.0-28.0)	26.0 (23.0-28.0)	27.0 (23.5-30.0)
**Estimated GFR (mL/min/1.73m^2^), median (Q1-Q3)**	56.9 (46.7-69.3)	56.9 (46.2-68.5)	56.9 (48.3-70.8)	63.6 (44.3-69.3)	56.2 (49.1-70.9)	59.4 (44.2-69.1)
**Thyroid stimulating hormone (mIE/L), median (Q1-Q3)**	1.5 (1.0-2.4)	1.5 (1.0-2.2)	1.6 (1.0-2.7)	1.5 (0.9-2.3)	1.6 (1.1-2.7)	1.7 (1.1-3.3)
**NT-proBNP (ng/L), median (Q1-Q3)**	390 (210-860)	340 (190-853)	445 (240-860)	250 (195-635)	470 (250-870)	680 (260-1925)
**Living in a nursing home, n (%)**	6 (1.8)	3 (1.9)	3 (1.9)	0	1 (0.9)	0
**Ever-smoker, n (%)**	77 (23.4)	32 (20.3)	43 (27.6)	11 (28.9)	32 (28.3)	2 (13.3)
**Atrial fibrillation on electrocardiogram, n (%)**	50 (15.2)	26 (16.5)	20 (12.8)	2 (5.3)	17 (15.0)	4 (26.7)
**Symptoms of OH, n (%)**	30 (9.1)	14 (8.9)	16 (10.3)	7 (18.4)	7 (6.2)	0
**Comorbidities, n (%)**						
Hypertension	178 (54.1)	83 (52.5)	86 (55.1)	18 (47.4)	66 (58.4)	9 (60.0)
Diabetes mellitus	58 (17.6)	25 (15.8)	29 (18.6)	5 (13.2)	24 (21.2)	4 (26.7)
Heart failure	57 (17.3)	34 (21.5)	19 (12.2)	5 (13.2)	12 (10.6)	4 (26.7)
Previous cardiovascular event	110 (33.4)	51 (32.3)	54 (34.6)	9 (23.7)	43 (38.1)	5 (33.3)
Neurological disease or dementia	53 (16.1)	29 (18.4)	24 (15.4)	7 (18.4)	16 (14.2)	0
**Blood pressure-lowering medications, n (%)**						
Renin-angiotensin-aldosterone system inhibitors	108 (32.8)	54 (34.2)	49 (31.4)	10 (26.3)	38 (33.6)	5 (33.3)
Calcium channel blockers	58 (17.6)	30 (19.0)	24 (15.4)	4 (10.5)	20 (17.7)	4 (26.7)
Beta-blockers	137 (41.6)	65 (41.1)	64 (41.0)	12 (31.6)	50 (44.2)	8 (53.3)
Loop diuretics	76 (23.1)	42 (26.6)	31 (19.9)	9 (23.7)	20 (17.7)	3 (20.0)
Thiazide diuretics	49 (14.9)	21 (13.3)	25 (16.0)	5 (13.2)	20 (17.7)	3 (20.0)
Spironolactone	16 (4.9)	10 (6.3)	6 (3.8)	0	6 (5.3)	0
**Other medications, n (%)**						
Nitrates^2^	55 (16.7)	18 (11.4)	34 (21.8)	5 (13.2)	27 (23.9)	3 (20.0)
Statins	81 (24.6)	31 (19.6)	46 (29.5)	11 (28.9)	34 (30.1)	4 (26.7)
Antidepressants	37 (11.2)	17 (10.8)	20 (12.8)	7 (18.4)	11 (9.7)	0
Sedatives (including sleeping medication)	60 (18.2)	32 (20.3)	27 (17.3)	7 (18.4)	19 (16.8)	1 (6.7)
**Health care contacts**						
Primary care (visits) (median (Q1-Q3)	5.0 (2.0-10.0)	4.0 (2.0-10.0)	5.0 (2.0-11.0)	4.0 (2.0-12.5)	5.0 (3.0-10.0)	5.0 (2.5-8.0)
Emergency department (visits), median (min-max)	0 (0-7)	0 (0-4)	0 (0-4)	0 (0-4)	0 (0-2)	0 (0-7)
Inpatient care (nights), median (min-max)	0 (0-33)	0 (0-59)	0 (0-13)	0 (0-59)	0 (0-11)	0 (0-33)
**Assessments of fall risk**						
Downton Fall Risk Index (points), median (Q1-Q3)^3^	4.0 (3.0-5.0)	4.0 (3.0-5.0)	4.0 (3.0-5.0)	3.0 (3.0-5.0)	4.0 (3.0-4.0)	3.0 (3.0-4.0)
Timed Up and Go (seconds), median (Q1-Q3)^3^	15.0 (12.0-20.0)	16.0 (13.0-20.0)	15.0 (12.0-22.0)	16.5 (12.0-23.8)	14.0 (12.0-19.0)	15.0 (11.3-17.8)
Downton Fall Risk Index ≥3 points, n (%)	271 (82.4)	128 (81.0)	131 (84.0)	31 (81.6)	96 (85.0)	12 (80.0)
Timed Up and Go ≥13.5 seconds, n (%)	201 (61.1)	99 (62.7)	94 (60.3)	25 (65.8)	64 (56.6)	8 (53.3)
**Assessments of functional ability, n (%)**						
EQ-5D (no difficulties)	163 (49.5)	84 (53.2)	73 (46.8)	17 (44.7)	55 (48.7)	6 (40.0)
IAM (fully independent)	59 (17.9)	31 (19.6)	25 (16.0)	5 (13.2)	20 (17.7)	3 (20.0)
PADL (fully independent)	282 (85.7)	138 (87.3)	130 (83.3)	31 (81.6)	97 (85.8)	14 (93.3)
**Assessments of cognitive functions, median (Q1-Q3)**						
Mini-Mental State Examination (points)	28.0 (26.0-29.0)	28.0 (27.0-29.0)	28.0 (26.0-29.0)	28.0 (26.0-29.0)	28.0 (26.0-29.0)	27.0 (26.0-29.0)
NYU-PRT, immediate (points)	5.0 (3.0-7.0)	5.0 (4.0-6.0)	5.0 (3.0-7.0)	5.0 (3.0-7.8)	5.0 (3.3-7.0)	5.0 (3.0-6.5)
NYU-PRT, delayed (points)	5.0 (3.0-7.8)	5.0 (3.0-7.0)	6.0 (3.0-8.0)	5.5 (2.0-7.3)	6.0 (3.0-8.0)	3.0 (3.0-7.5)
30-item Naming test (points)	24.0 (21.0-27.0)	23.0 (20.0-27.0)	25.0 (21.0-27.0)	25.0 (21.3-27.0)	25.0 (21.0-27.0)	23.0 (21.5-25.5)
Symbol Digit Modalities Test (points)	21.0 (16.0-27.0)	21.0 (15.8-27.0)	22.0 (17.0-28.0)	25.0 (18.0-29.0)	22.0 (17.0-27.0)	19.0 (13.5-25.5)
CLOX and Cube Copying Test (points)	10.0 (9.0-12.0)	10.0 (9.0-12.0)	10.0 (8.0-12.0)	10.0 (8.0-11.0)	10.0 (9.0-12.0)	10.0 (9.5-11.0)
Token Test short version (points)	5.0 (4.0-6.0)	5.0 (4.0-6.0)	5.0 (4.0-6.0)	4.5 (4.0-5.0)	5.0 (3.8-6.0)	5.0 (2.5-5.0)
The PaSMO (seconds)^3^	100.0 (77.0-135.0)	107.0 (78.0-149.0)	96.5 (72.3-123.8)	95.0 (71.8-145.8)	97.5 (73.8-119.0)	105.0 (75.5-116.3)
The Rey-Complex Figure Test (points)	17.0 (16.0-18.0)	17.0 (16.0-18.0)	17.0 (16.0-18.0)	17.0 (15.0-18.0)	17.0 (16.0-18.0)	17.0 (16.0-18.0)
Victoria Stroop Test 1 (seconds)^3^	19.0 (16.0-24.0)	19.0 (15.0-24.0)	19.0 (16.8-23.3)	19.0 (16.0-21.0)	19.0 (17.0-24.8)	22.0 (17.5-22.5)
Victoria Stroop Test 2 (seconds)^3^	31.0 (24.0-39.0)	31.0 (23.8-37.3)	31.5 (24.0-40.0)	30.0 (24.0-41.0)	32.0 (25.0-39.0)	31.0 (24.5-38.5)
Victoria Stroop Test 3 (seconds)^3^	45.0 (36.0-60.5)	44.0 (36.0-59.0)	46.5 (36.3-63.0)	49.5 (40.0-63.0)	45.0 (34.0-62.5)	49.0 (39.0-69.0)
Trail Making Test A (seconds)^3^	65.0 (51.0-90.0)	64.0 (51.0-88.0)	65.0 (51.0-90.0)	61.0 (51.5-77.5)	67.0 (51.0-92.0)	67.0 (55.5-109.0)

1 Includes 5 participants with classical OH that could not be classified as transient or continuous because of missing values at 5 and 10 minutes of standing up.

2 Nitrates includes both short-acting sublingual preparations and long-acting oral preparations.

3 Lower score implies better cognitive function. For the other tests, higher score implies better cognitive function. CLOX, The Clock Drawing Task; GFR, glomerular filtration rate; IAM, Instrumental activity measure; NT-proBNP, N-terminal prohormone of brain natriuretic peptide; NYU-PRT, New York University Paragraph recall test; OH, orthostatic hypotension; PADL, Personal activities of daily living; PaSMO, Parallel serial mental operations.

**Figure 2.**
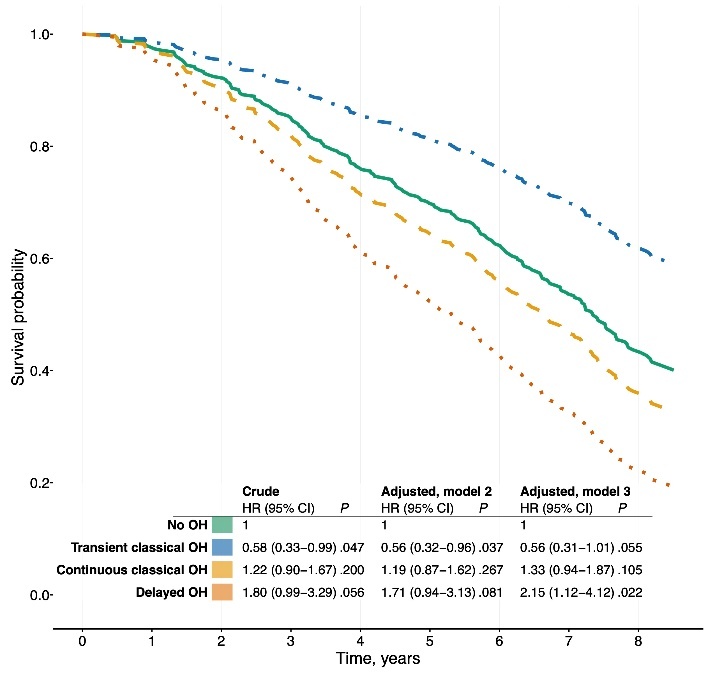
**Cumulative survival probability after a follow-up time of 8.6 years for participants depending on orthostatic hypotension status at baseline: no (solid green line), transient classical (dash-dotted blue line), continuous classical (dashed yellow line), or delayed OH (dotted orange line)**. Abbreviations: HR, hazard ratio; OH, orthostatic hypotension.

### Comparisons at baseline

Of the included participants, 191 (58.1%) were women. There were fewer women amongst those with delayed vs no OH, 5 (33.3%) vs 99 (62.7%), *P* = .027. Of participants, 178 (54.1%) had hypertension, 57 (17.3%) had heart failure, and 53 (16.1%) had neurological disease or dementia. Heart failure was less common amongst those with classical vs no OH, 19 (12.2%) vs 34 (21.5%), *P* = .027. Of the participants with delayed vs no OH, none vs 29 (18.4%) had neurological disease or dementia, *P* = .070, Table 1.

Of all participants, 30 (9.1%) had symptoms of OH, and those with transient classical OH had symptoms more often, OR (95% CI) 2.77 (0.86-8.91), *P* = .088 after multiple adjustment. Participants with continuous classical and delayed vs no OH had higher systolic supine BP, mean (95% CI) 147.0 (133.9-160.1) and 154.2 (138.0-170.4) vs 139.6 (127.0-152.3) mmHg, *P* = .005 and .009, respectively, after multiple adjustment. The use of nitrates was more common amongst those with continuous classical vs no OH, OR (95% CI) 3.38 (1.53-7.50), *P* = .003 after multiple adjustment, Table 2, and Supplementary Tables 2 and 3.

Assessments of functional ability and fall risk did not differ between participants according to OH status. In cognitive function assessments, participants with continuous classical vs no OH had higher scores in the 30-item Naming Test, mean (95% CI) 21.6 (18.3-24.9) vs 20.3 (17.1-23.4) points, *P* = .048, and were slower in the Parallel Serial Mental Operations test, mean (95% CI) 124.1 (72.4-175.9) vs 139.0 (89.5-188.5) seconds, *P* = .042, both after multiple adjustments. Participants with continuous classical vs no OH had numerically lower scores in the Clock Drawing Task and Cube Copying Test, mean (95% CI) 8.3 (6.9-9.7) vs 8.8 (7.5-10.2) points, *P* = .057, and participants with delayed vs no OH had lower scores in the Token Test short version, mean (95% CI) 3.2 (1.9-4.5) vs 4.0 (2.9-5.1) points, *P* = .065, both after multiple adjustments, Table 2.

**Table 2:** The odds ratios, means and 95% CI for the baseline results in relation to orthostatic hypotension status.

	No OH, n = 158	Classical OH		Delayed OH, n = 15
		Transient, n = 38	Continuous, n = 113	
	OR (95% CI)	OR (95% CI)	OR (95% CI)	OR (95% CI)
**Living in a nursing home**	1	NA^1^	0.46 (0.05-4.49)	NA^1^
**Neurological disease or dementia**	1	1.00 (0.40-2.50)	0.73 (0.38-1.43)	NA^1^
**Atrial fibrillation on electrocardiogram**	1	0.28 (0.06-1.25)	0.89 (0.46-1.73)	1.75 (0.52-5.92)
**Elevated supine blood pressure**	1	0.94 (0.45-1.99)	2.28 (1.27-4.10)	6.87 (0.88-53.66)
**Abnormal pulse response**	1	1.32 (0.61-2.85)	1.23 (0.72-2.10)	0.44 (0.10-2.03)
**Symptoms of OH**	1	2.32 (0.87-6.23)	0.68 (0.26-1.74)	NA^1^
**Blood pressure-lowering medications**				
**Renin-angiotensin-aldosterone system inhibitors**	1	0.69 (0.31-1.52)	0.98 (0.59-1.63)	0.96 (0.31-2.96)
**Calcium channel blockers**	1	0.50 (0.16-1.51)	0.91 (0.49-1.70)	1.54 (0.46-5.17)
**Beta-blockers**	1	0.65 (0.31-1.39)	1.12 (0.69-1.83)	1.62 (0.56-4.68)
**Loop diuretics**	1	0.85 (0.37-1.94)	0.59 (0.32-1.07)	0.68 (0.18-2.55)
**Thiazide diuretics**	1	0.98 (0.34-2.80)	1.39 (0.71-2.71)	1.62 (0.42-6.22)
**Spironolactone**	1	NA^1^	0.82 (0.29-2.34)	NA^1^
**Other medications**				
**Nitrates^2^**	1	1.17 (0.40-3.38)	2.42 (1.26-4.66)	1.93 (0.50-7.50)
**Statins**	1	1.66 (0.74-3.70)	1.75 (1.00-3.07)	1.48 (0.44-4.96)
**Antidepressants**	1	1.86 (0.71-4.87)	0.89 (0.40-1.98)	NA^1^
**Sedatives (incl. sleeping medication)**	1	0.88 (0.36-2.19)	0.79 (0.42-1.48)	0.28 (0.04-2.20)
**Polypharmacy (≥5 medications)**	1	0.76 (0.37-1.55)	1.15 (0.70-1.87)	1.27 (0.43-3.74)
**Assessments of fall risk**				
**Downton Fall Risk Index ≥3 points^3^**	1	1.04 (0.42-2.58)	1.32 (0.69-2.54)	0.94 (0.25-3.53)
**Timed Up and Go ≥13.5 seconds^3^**	1	1.09 (0.52-2.29)	0.80 (0.49-1.33)	0.75 (0.25-2.28)
**Assessments of functional ability**				
**EQ-5D (no difficulties)**	1	0.75 (0.37-1.54)	0.85 (0.52-1.38)	0.59 (0.20-1.73)
**PADL (fully independent)**	1	0.64 (0.25-1.65)	0.88 (0.43-1.78)	2.03 (0.25-16.28)
**IAM (fully independent)**	1	0.44 (0.14-1.35)	0.77 (0.37-1.59)	0.74 (0.16-3.32)
	Mean (95% CI)	Mean (95% CI)	Mean (95% CI)	Mean (95% CI)
**Supine blood pressure (mmHg)**				
**Systolic**	148.1 (144.7-151.4)	149.0 (142.2-155.8)	155.9 (152.0-159.9)	161.0 (150.2-171.8)
**Diastolic**	71.4 (69.8-73.0)	72.9 (69.7-76.2)	76.1 (74.2-78.0)	80.9 (75.7-86.0)
**Health care contacts**				
**Primary care (visits)**	8.5 (6.2-10.7)	9.2 (4.6-13.8)	10.5 (7.8-13.2)	7.9 (0.5-15.2)
**Emergency department (visits)**	0.4 (0.3 to 0.6)	0.4 (0.1 to 0.7)	0.5 (0.3 to 0.6)	0.4 (-0.0 to 0.8)
**Inpatient care (nights)**	1.6 (0.8 to 2.4)	1.0 (-0.6 to 2.6)	1.5 (0.5 to 2.4)	0.9 (-1.6 to 3.5)
**Assessments of fall risk**				
**Downton Fall Risk Index (points)^3^**	3.7 (3.4-3.9)	3.8 (3.3-4.3)	3.7 (3.4-4.0)	3.3 (2.6-4.1)
**Timed Up and Go (seconds)^3^**	17.1 (16.1-18.2)	18.8 (16.7-21.0)	16.6 (15.3-17.8)	14.8 (11.2-18.3)
**Assessments of cognitive functions**				
**Mini-Mental State Examination (points)**	27.4 (26.9-27.9)	26.4 (25.4-27.5)	27.3 (26.7-27.9)	27.1 (25.4-28.7)
**NYU-PRT, immediate (points)**	5.2 (4.7-5.6)	5.1 (4.2-6.0)	5.4 (4.9-5.9)	5.3 (3.9-6.8)
**NYU-PRT, delayed (points)**	5.4 (4.8-5.9)	5.2 (4.1-6.2)	5.6 (5.0-6.2)	4.8 (3.2-6.4)
**30-item Naming test (points)**	22.2 (21.4-23.1)	23.4 (21.8-25.1)	23.7 (22.8-24.7)	23.3 (20.6-25.9)
**Symbol Digit Modalities Test (points)**	21.5 (20.1-22.9)	22.8 (19.9-25.7)	22.5 (20.8-24.2)	19.5 (15.1-23.9)
**CLOX and Cube Copying test (points)**	10.1 (9.8-10.5)	9.2 (8.5-9.9)	9.7 (9.3-10.1)	10.1 (9.0-11.2)
**Token Test short version (points)**	4.6 (4.3-4.8)	4.3 (3.8-4.7)	4.5 (4.2-4.7)	3.9 (3.1-4.6)
**The PaSMO (seconds)^3^**	115.7 (107.5-124.0)	111.3 (92.8-129.8)	103.2 (93.3-113.1)	104.6 (72.6-136.7)
**The Rey-complex Figure test (points)**	16.4 (15.9-16.9)	15.9 (15.0-16.8)	16.2 (15.7-16.8)	16.2 (14.7-17.6)
**Victoria Stroop Test 1 (seconds)^3^**	22.6 (20.2-24.9)	22.2 (17.5-26.9)	22.1 (19.2-24.9)	21.3 (13.8-28.8)
**Victoria Stroop Test 2 (seconds)^3^**	33.7 (31.0-36.4)	36.7 (31.2-42.1)	35.8 (32.6-39.0)	31.8 (23.4-40.2)
**Victoria Stroop Test 3 (seconds)^3^**	50.4 (46.1-54.7)	57.2 (48.6-65.9)	52.8 (47.7-57.9)	61.0 (47.6-74.4)
**Trail Making Test A (seconds)^3^**	76.3 (69.9-82.6)	72.1 (59.2-85.1)	75.6 (67.9-83.2)	85.4 (65.6-105.2)

1 No occurrences.

2 Nitrates includes both short-acting sublingual preparations and long-acting oral preparations.

3 Lower score implies better cognitive function. For the other tests, higher score implies better cognitive function. CLOX, The Clock Drawing Task; IAM, Instrumental Activity Measure; NYU-PRT, New York University Paragraph Recall Test; OH, orthostatic hypotension; PADL, Personal Activities of Daily Living; PaSMO, Parallel Serial Mental Operations.

**Table 3:** Mean (95% CI) change in assessments of cognitive functions between baseline and follow-up after 5 years for participants with classical vs no orthostatic hypotension at baseline.

	No OH, n = 55	Classical OH, n = 54			
	Mean change (95% CI)	Mean change (95% CI)	Crude, *P*	Model 2, *P*	Model 3, *P*
**Mini-Mental State Examination (points)**	-1.9 (-2.8 to -0.9)	-1.9 (-3.0 to -0.8)	.970	.594	.439
**NYU-PRT, immediate (points)**	-0.4 (-1.7 to 0.8)	-0.1 (-1.3 to 1.1)	.657	.447	.375
**NYU-PRT, delayed (points)**	-0.5 (-1.8 to 0.8)	-0.2 (-1.6 to 1.2)	.715	.438	.350
**30-item Naming test (points)**	-0.9 (-2.6 to 0.8)	-2.4 (-4.0 to -0.9)	.148	.158	.243
**Symbol Digit Modalities Test (points)**	-3.5 (-6.5 to -0.4)	-3.3 (-6.2 to -0.3)	.924	.961	.975
**CLOX and Cube Copying Test (points)**	-1.1 (-2.0 to -0.2)	-0.8 (-1.6 to 0.0)	.615	.662	.648
**Token Test short version (points)**	0.0 (-0.5 to 0.5)	-0.3 (-0.8 to 0.3)	.363	.372	.328
**The PaSMO (seconds)^1^**	20.6 (-6.5 to 47.8)	16.9 (-14.5 to 48.2)	.833	.926	.746
**The Rey-complex Figure test (points)**	-0.8 (-2.1 to 0.4)	-1.3 (-2.7 to 0.2)	.650	.762	.380
**Victoria Stroop Test 1 (seconds)^1^**	9.7 (0.1 to 19.4)	6.6 (-1.3 to 14.5)	.614	.476	.564
**Victoria Stroop Test 2 (seconds)^1^**	9.1 (-0.2 to 18.5)	10.3 (2.5 to 18.0)	.857	.772	.799
**Victoria Stroop Test 3 (seconds)^1^**	9.5 (-2.5 to 21.5)	5.8 (-5.5 to 17.0)	.667	.558	.572
**Trail Making Test A (seconds)^1^**	23.2 (-2.1 to 48.4)	15.6 (-9.5 to 40.7)	.644	.623	.638

1 Lower score implies better cognitive function. For the other tests, higher score implies better cognitive function. CLOX, The Clock Drawing Task; NYU-PRT, New York University Paragraph Recall Test; OH, orthostatic hypotension; PaSMO, Parallel Serial Mental Operations.

### Results at follow-up

After 5 years, 111 (33.7%) of the participants remained in the study, Supplementary Figure 1. Of these, 54 (48.6%) had classical OH, 55 (49.5%) had no OH, and 2 (1.8%) had delayed OH at baseline, not shown. Because of the very few participants, those with delayed OH were not included, and classical OH was not subcategorized, in these analyses. The mean change in assessments of cognitive functions were numerically either unchanged, negative (for analyses in which higher score denotes better performance), or positive (for analyses in which lower score denotes better performance), but there were no differences in relation to OH status, Table 3.

### All-cause mortality

After 8.6 years, 195 (59.3%) of the participants had died, of which 92 (58.2%), 15 (39.5%), 72 (63.2%), and 12 (80%) had no, transient classical, continuous classical, and delayed OH at baseline, respectively. The remaining 4 (2.1%) had classical OH which could not be defined further. Delayed vs no OH was associated with twice the risk of all-cause mortality, *P* = .056 in the crude analysis, and HR 2.15 (95% CI 1.12-4.12), *P* = .022, after multiple adjustments. Transient classical vs no OH was associated with half the risk of all-cause mortality when adjusted for sex, HR 0.56 (95% CI 0.32-0.96), *P* = .037, but this was no longer significant after multiple adjustments, HR 0.56 (95% CI 0.31-1.01), *P* = .055, Figure 2.

## DISCUSSION

This is the first study of classical and delayed OH in a general population sample of 85-year-olds. Of participants, almost half had classical OH, and only 4.6% had delayed OH. Participants with delayed OH had twice the mortality risk in the following 8.6 years when compared to those without OH.

### Prevalence

The prevalence of classical OH was higher than in most previous studies. In 7 studies of individuals with a mean age of 72-81 years, the prevalence of classical OH was 7% (primary care patients), 18%, 21.5%, and 27% (general population cohorts), 35% (geriatric patients), as well as 38.5% and 50% (hospitalized patients) [12-18]. However, the first 3 studies measured BP only at 3 minutes, and the fourth study used only systolic BP, which could decrease sensitivity [16-18]. A Dutch study of patients with falls and syncope and a mean age of 80 years, which measured standing BP after 1, 3, 5, 7, 9, and 10 minutes, found that 32% had continuous classical, 16% had transient classical, and 8% had delayed OH [19]. The prevalence may also depend on age, as classical OH has consistently increased with age in previous studies [16, 17, 20-26].

The prevalence of delayed OH in the general population is not known [3]. The 8% prevalence found in the Dutch cohort referenced above is somewhat higher than in our study [19]. In an Israeli study of admitted patients with a mean age of 76 years, 14.8% of patients had delayed OH, but standing BP was measured at 1, 3, and 5 (and not 10) minutes [14]. Sensitivity for OH may increase substantially when standing BP measurements are performed for longer time periods [27], but consensus is lacking regarding the duration of standing BP measurements. Finally, an association between delayed OH and mortality could also result in an increased ratio between classical vs delayed OH with age.

### Association with blood pressure, antihypertensives, functional ability, and risk of falls

The systolic and diastolic supine BP was higher in participants with continuous classical and delayed vs no OH, which has also been seen in several previous studies [12, 14-17, 19, 21-24, 26, 28]. Although taking nitrates at baseline was more common in those with continuous classical OH, OH status was not associated with assessments of functional ability or fall risk. In previous studies, classical OH has been associated with slower gait speeds, difficulty walking, increased incidence of falls, lower Instrumental Activities of Daily Living score, and slower performance in the Timed Up and Go test [12, 15, 16, 29, 30]. However, all those cohorts had a mean age below 80 years. The overall fall risk at baseline in this cohort was rather high (e.g., 82.4% according to the Downton Fall Risk Index), and may suggest that OH has less impact on fall risk in these older individuals, or that other evaluations including the incidence of actual falls, or a larger sample size, may be necessary to reveal differences between groups.

### Cognitive assessments and the prevalence of neurodegenerative disease

Most cognitive assessments at baseline did not differ depending on OH status. However, participants with continuous classical OH performed better in the 30-item Naming Test, but worse in the Parallel Serial Mental Operations test. Other observed differences between groups were numerical or did not remain after multiple adjustment. Finally, during the 5-year follow-up, results of all tests declined regardless of OH status. Several previous studies, including two meta-analyses, have found associations between classical OH and neurodegenerative disorders, including in individuals aged in their 70’s and 80’s [12, 28, 31, 32]. Delayed OH was associated with neurodegenerative disease in a study of admitted patients [14]. Four previous studies also found that individuals with classical OH scored lower on Mini-Mental State Examination, but the patients in two of these studied had known cognitive symptoms and Parkinson’s disease, respectively [16, 17, 22, 33]. The current study may have been less sensitive to differences between groups because of the non-patient cohort and the high rate of loss to follow-up.

### All-cause mortality

Delayed, but not classical OH, was associated with all-cause mortality. This is contrary to previous studies, in which classical vs delayed OH has been more strongly associated with mortality [3]. However, in a recent meta-analysis of 13 studies, the association between classical OH and all-cause mortality was more pronounced amongst those under 65 years [4]. Our results may imply that this age factor is decisive. Furthermore, transient classical OH was associated with decreased all-cause mortality in the crude analysis, but not after multiple adjustments. Similarly in a Dutch study, the mortality rate for transient vs continuous classical OH was numerically lower at 5% vs 14% after a 2.5 year follow-up, and for delayed OH numerically higher at 17% [19]. If the current results are replicated, it would indicate that standing BP measurements beyond 3 minutes are valuable even when classical OH has been confirmed, and that delayed OH may have different implications in 85-year-olds vs younger age groups.

### Limitations

To the best of our knowledge this is the first study of 85-year-olds, with a follow-up time of more than 5 years, which includes both classical and delayed OH. All the participants had the same age at inclusion, and outcomes could be adjusted for many impactful co-morbidities. However, almost half of those invited declined to participate or were lost to follow-up before the first BP measurements. Compared with participants, those that declined were more often women (69.8% vs 58.1%, *P* = .002), and nursing home residents (24.6% vs 6.4%, *P* <.001), and had more yearly in-patient care days (mean 4.0 vs 1.5, *P* = .003). Thus, the studied cohort was somewhat healthier than the invited population. The participants were from a midsize city in Sweden, and thus generalizability outside this context cannot be assumed. BP was not measured after 10 minutes, and thus some cases of delayed OH may have been missed [34]. Medical history and medications were retrieved from questionnaires rather than registry data, thus could include inaccuracies. Multiple testing can raise the concern of false positives, and our results should thus be interpreted with caution. Some results in this study are novel and will thus need to be replicated. Finally, as with previous studies, whether OH is causative and warrants targeted interventions or merely is a marker of comorbidities, cannot be determined from this study [3-5].

### Conclusion

In this prospective study of 329 women and men aged 85 years, 47.4% had classical and 4.6% had delayed OH. Participants with OH, whether classical or delayed, had no increased risk of cognitive decline. Delayed OH was independently associated with increased mortality, and transient classical OH was associated with reduced mortality. This indicates that OH may have different implications for morbidity and mortality in 85-year-olds compared with younger populations.

## Supplementary Materials

The Supplementary data can be found online at: www.aginganddisease.org/EN/10.14336/AD.2024.0205.


