# Supplementary material for: Orthostatic Hypotension and Cognitive Function in Individuals 85 Years of Age: A Longitudinal Cohort Study in Sweden
Source: Aging Dis. 2024 Feb 5;16(1):469–78. doi: 10.14336/AD.2024.0205 (PMC11745430; doi:10.14336/AD.2024.0205)
Supplement: Supplementary file 1 [file AD-16-1-469-s.pdf]

Orthostatic Hypotension and Cognitive Function in Individuals 85 Years of Age: A Longitudinal Cohort Study in Sweden

Peder af Geijerstam, Katie Harris, Maria M. Johansson, John Chalmers, Katarina Nägga, Karin Rådholm

© 2024. af Geijerstam P et al. Published online at http://www.aginganddisease.org/EN/10.14336/AD.2024.0205

SUPPLEMENTARY MATERIAL

Methods, baseline measurements

Body mass index (BMI) was calculated as weight (kg) divided by the square of height (m). Thyroid stimulating hormone, N-terminal prohormone of brain natriuretic peptide (NT-proBNP) and serum creatinine were analyzed from non-fasting venous blood samples as previously described in detail [1]. Estimated glomerular filtration rate (eGFR) was calculated from serum creatinine, age, and sex using the 2021 Chronic Kidney Disease Epidemiology Collaboration (CKD-EPI) equation [2].

The presence of atrial fibrillation was assessed manually according to the American Heart Association (AHA) guidelines from a standard 12 lead electrocardiogram recorded at 50 mm/s and 10 mm/mV [1]. The prevalence of other comorbidities was derived from the baseline questionnaire.

Methods, Blood pressure and pulse measurements

Supine BP was categorized as elevated if systolic BP was at or above 140 mmHg and/or diastolic BP was at or above 90 mmHg, and normal if both measurements were below those levels.

The maximum decrease of BP within the first 3 minutes was calculated by subtracting the supine BP from the BP at 1 and 3 minutes, respectively, and then selecting the smallest of these two differences. The maximum decrease of BP within 5 to 10 minutes was calculated by subtracting the supine BP from the BP at 5 and 10 minutes, respectively, and then selecting the smallest of these two differences. If any of the BP measurements after 3, 5, or 10 minutes of standing up were missing, the BP measurements obtained during the procedure were used to select the maximum decrease of BP.

The maximum pulse increase was calculated by subtracting the pulse in the supine position from the pulse at 1, 3, 5, and 10 minutes, respectively, and then selecting the largest of these four differences. This value was then categorized as normal if higher than 10 beats per minute, and abnormal if less than or equal to 10 beats per minute.

Methods, assessments of fall risk and functional ability

The Downton Fall Risk Index (DFRI) questionnaire comprised of 11 questions: previous falls, 5 different medications, 3 different sensory deficits, mental state, and gait. For each of these that were present, the score 1 was given, and the total score was between 0 and 11. In a Swedish study evaluating over 6000 patients with a mean age of 84 years, the DFRI was associated with fall-related injuries.[3] In the Timed Up and Go test, the individuals were asked to rise from an armchair, walk 3 meters, and then return to the chair and sit back down. The score was the number of seconds it took the participants to complete the task.

The EQ-5D questionnaire is comprised of 5 subitems of which 3 were used in this study: mobility, self-care, and usual activities. The response options for mobility were "I have no problems in walking about", "I have some problems in walking about", and "I am confined to bed"; for self-care were "I have no problems with self-care", "I have some problems washing or dressing myself", and "I am unable to wash or dress myself"; and for usual activities were "I have no problems with performing my usual activities", "I have some problems with performing my usual activities", and "I am unable to perform my usual activities".

The Instrumental Activity Measure (IAM) questionnaire covered 8 activities: locomotion outdoors, preparing a simple meal, cooking, using public transport, small-scale shopping, large-scale shopping, cleaning, and laundry. The response options were "no difficulties", "some difficulties", "great difficulties, "too difficult", and "I have not tried/not applicable". The latter response option was treated as missing.

The Personal Activities of Daily Living (PADL) questionnaire covered 4 activities: bathing/showering, dressing and undressing, toileting, and eating. For each activity, participants were asked if they could manage it independently, and response options were "manages without help", "I need some help", and "I need much help".

Methods, statistical analyses

In multiple adjustments, Model 3, living in a nursing home was not included when analyzing living in a nursing home, presence of neurological disease or dementia was not included when analyzing presence of neurological disease or dementia, atrial fibrillation was not included when analyzing atrial fibrillation, supine BP was not included when analyzing elevated supine BP or abnormal pulse response, use of

© 2024. af Geijerstam P et al. Published online at http://www.aginganddisease.org/EN/10.14336/AD.2024.0205

antidepressants were not included when analyzing use of antidepressants, use of sedatives were not included when analyzing use of sedatives, and polypharmacy was not included when analyzing polypharmacy.

Data missing completely at random or at a rate below 10% were managed by listwise deletion, data missing at random at a rate above 20% were managed by multivariate imputations by chained equations (R package "mice" version 3.16.0) with a setting of 20 imputations and 20 iterations [4].

Results, missingness

Standing BP measurements were not obtained from 3, 5, and 10 minutes and onwards for 3 (0.9%), 2 (0.6%), and 10 (3.0%) of the participants, respectively. Based on the preceding standing BP measurements, all of these were categorized as having classical OH, except for 5 of those with missing values at 10 minutes, which were classified as having no OH. Missing rate for all other variables at baseline was less than 10%, except for the Parallel Serial Mental Operations test for which the missing rate was 27.4%, and these were managed using listwise deletion. At follow-up, the missing rates amongst the 109 participants that had not been lost to follow-up were 20-30% for the Clock Drawing Task and Cube Copying Test, the Token Test short version, the Symbol Digit Modalities Test, the Rey-Complex Figure Test, the Victoria Stroop Tests, and the Trail Making Test A, and 43% for the Parallel Serial Mental Operations test. These were considered as missing at random and managed using multivariate imputations by chained equations.

References

[1] Rådholm K, Östgren CJ, Alehagen U, Falk M, Wressle E, Marcusson J *et al. Atrial fibrillation (AF) and co-morbidity in elderly. A population based survey of 85 years old subjects*. Arch Gerontol Geriatr 2011;**52**:e170-5.

[2] Inker LA, Eneanya ND, Coresh J, Tighiouart H, Wang D, Sang Y *et al. New Creatinine- and Cystatin C-Based Equations to Estimate GFR without Race*. N Engl J Med 2021;**385**:1737-49.

[3] Mojtaba M, Alinaghizadeh H, Rydwik E. *Downton Fall Risk Index during hospitalisation is associated with fall-related injuries after discharge: a longitudinal observational study*. J Physiother 2018;**64**:172-77.

[4] van Buuren S, Groothuis-Oudshoorn K, Vink G, Schouten R, Robitzsch A, Rockenschaub P *et al. mice: Multivariate Imputation by Chained Equations.* https://cran.r-project.org/web/packages/mice/index.html (2023-09-24 date last accessed).

© 2024. af Geijerstam P et al. Published online at http://www.aginganddisease.org/EN/10.14336/AD.2024.0205

Supplementary Table 1. Blood pressure and pulse measurements in the supine position and after 1, 3, 5, and 10 minutes of standing up, respectively, in relation to orthostatic hypotension status at baseline, shown as the mean and 95% CI.

	**All participants, N = 329**	**No OH, n = 158**	**Classical OH**			**Delayed OH, n = 15**
			**All**[1], n = 156	**Transient,** n = 38	**Continuous,** n = 113	
**Systolic BP(mmHg), mean (95% CI)**						
Supine	151.6 (149.3-154.0)	148.1 (144.5-151.7)	154.3 (151.2-157.5)	149.0 (142.3-155.8)	155.9 (152.3-159.5)	161.0 (149.0-173.0)
After 1 min of standing up	140.7 (138.1-143.4)	147.9 (144.2-151.7)	132.2 (128.6-135.9)	128.7 (119.9-137.4)	133.6 (129.7-137.5)	153.9 (142.7-165.1)
After 3 min of standing up	142.2 (139.6-144.9)	149.5 (145.8-153.3)	133.7 (130.2-137.2)	138.2 (130.8-145.5)	131.9 (128.1-135.8)	153.0 (140.3-165.7)
After 5 min of standing up	142.0 (139.4-144.6)	149.1 (145.3-152.9)	134.2 (130.8-137.6)	141.9 (134.9-148.9)	131.6 (127.8-135.4)	145.3 (133.5-157.2)
After 10 min of standing up	144.4 (141.7-147.2)	151.1 (147.1-155.1)	137.3 (133.7-140.9)	146.3 (138.1-154.5)	134.2 (130.4-138.1)	146.0 (132.9-159.1)
Max decrease vs supine within 1-3 min	-14.1 (-15.8 to -12.3)	-2.2 (-3.6 to -0.9)	-26.4 (-28.3 to -24.4)	-21.9 (-25.2 to -18.6)	-27.3 (-29.7 to -25.0)	-10.1 (-12.6 to -7.6)
Max decrease vs supine within 5-10 min	-11.8 (-13.6 to -10.1)	-1.1 (-2.7 to 0.5)	-22.3 (-24.6 to -20.0)	-8.9 (-11.0 to -6.8)	-26.8 (-29.2 to -24.3)	-19.3 (-26.6 to -12.0)
**Diastolic BP (mmHg), mean (95% CI)**						
Supine	73.8 (72.6-74.9)	71.4 (69.8-73.1)	75.4 (73.8-77.0)	72.9 (69.8-76.1)	76.1 (74.2-77.9)	80.9 (75.8-86.0)
After 1 min of standing up	74.3 (72.8-75.8)	76.0 (73.6-78.4)	72.0 (70.1-73.9)	71.1 (66.3-75.8)	72.0 (70.0-74.0)	81.7 (75.2-88.1)
After 3 min of standing up	74.3 (73.0-75.5)	75.3 (73.5-77.1)	72.8 (70.9-74.6)	74.3 (70.5-78.2)	71.7 (69.6-73.8)	78.6 (72.9-84.3)
After 5 min of standing up	74.3 (73.1-75.6)	75.6 (73.8-77.4)	73.0 (71.1-75.0)	76.3 (72.0-80.6)	71.9 (69.7-74.1)	74.3 (68.5-80.0)
After 10 min of standing up	75.4 (74.1-76.6)	76.2 (74.4-78.1)	74.2 (72.4-76.1)	78.5 (74.5-82.5)	72.8 (70.8-74.8)	77.3 (70.8-83.9)
Max decrease vs supine within 1-3 min	-1.5 (-2.3 to -0.6)	2.3 (1.4 to 3.2)	-5.2 (-6.5 to -4.0)	-3.2 (-5.8 to -0.7)	-6.1 (-7.6 to -4.7)	-1.2 (-4.1 to 1.7)
Max decrease vs supine within 5-10 min	-0.6 (-1.5 to 0.2)	2.6 (1.6 to 3.7)	-3.5 (-4.8 to -2.2)	2.7 (0.8 to 4.7)	-5.6 (-7.0 to -4.2)	-6.9 (-10.8 to -3.0)
**Pulse (beats/minute), mean (95% CI)**						
Supine	67.1 (65.9-68.4)	67.9 (66.1-69.8)	66.5 (64.7-68.3)	69.1 (65.4-72.9)	65.5 (63.3-67.6)	65.1 (59.2-71.0)
After 1 min of standing up	73.1 (71.7-74.4)	73.4 (71.3-75.5)	73.1 (71.1-75.0)	77.1 (73.0-81.2)	71.7 (69.5-73.9)	69.6 (63.7-75.5)
After 3 min of standing up	72.1 (70.7-73.4)	72.2 (70.1-74.3)	72.2 (70.3-74.1)	76.2 (72.3-80.1)	70.8 (68.6-72.9)	68.3 (61.7-74.8)
After 5 min of standing up	72.0 (70.6-73.3)	72.5 (70.4-74.6)	71.7 (69.8-73.7)	74.9 (70.9-79.0)	70.7 (68.5-72.9)	68.1 (62.3-74.0)
After 10 min of standing up	72.7 (71.3-74.2)	73.4 (71.1-75.7)	72.4 (70.3-74.4)	77.2 (72.8-81.7)	70.7 (68.4-73.0)	70.0 (63.6-76.4)
Max increase vs supine within 1-3 min	6.7 (6.1-7.3)	6.1 (5.2-7.0)	7.4 (6.5-8.4)	8.8 (7.2-10.4)	7.1 (5.9-8.2)	5.3 (3.3-7.4)
Max increase vs supine within 5-10 min	6.7 (6.0-7.4)	6.5 (5.4-7.6)	7.1 (6.0-8.1)	8.5 (6.3-10.7)	6.6 (5.3-7.8)	5.6 (3.4-7.8)
**OH categorization vs BP**[2]**, n (%)**						
Isolated systolic OH	63 (19.1)	NA	81 (51.9)	25 (65.8)	52 (46.0)	5 (33.3)
Isolated diastolic OH	86 (26.1)	NA	17 (10.9)	5 (13.2)	12 (10.6)	5 (33.3)
Combined systolic and diastolic OH	22 (6.7)	NA	58 (37.2)	8 (21.1)	49 (43.4)	5 (33.3)
**Systolic BP drop <90 mmHg**[3]**, n (%)**	4 (1.2)	0	4 (2.6)	1 (2.6)	3 (2.7)	0

1 Includes 5 participants with classical OH that could not be classified as transient or continuous because of missing values at 5 and 10 minutes of standing up.
2 OH was categorized as isolated systolic OH if systolic standing BP dropped at least 20 mmHg at 1, 3, 5, and/or 10 minutes; isolated diastolic OH if diastolic standing BP dropped at least 10 mmHg at 1, 3, 5, and/or 10 minutes; and as combined systolic; and diastolic OH if both of these criteria were fulfilled (not necessarily at the same point in time).
3 This was evaluated for each of 1, 3, 5, and 10 minutes of standing systolic BP, and if it dropped below 90 mmHg at any or several of these measurements, it was categorized as such.
BP, blood pressure; OH, orthostatic hypotension.

Supplementary Table 2. The odds ratios, means and 95% CI for the baseline results in relation to orthostatic hypotension status after adjustments of sex (model 2).

	**No OH,** n = 158	**Classical OH**		**Delayed OH,** n = 15
		**Transient,** n = 38	**Continuous,** n = 113	
	**OR (95% CI)**	**OR (95% CI)**	**OR (95% CI)**	**OR (95% CI)**
**Living in a nursing home**	1	NA[1]	0.49 (0.05-4.85)	NA[1]
**Neurological disease or dementia**	1	1.00 (0.40-2.49)	0.71 (0.37-1.39)	NA[1]
**Atrial fibrillation on electrocardiogram**	1	0.28 (0.06-1.25)	0.84 (0.43-1.65)	1.52 (0.44-5.24)
**Elevated supine blood pressure**	1	0.95 (0.45-2.01)	2.36 (1.31-4.26)	7.51 (0.95-59.14)
**Abnormal pulse response**	1	1.31 (0.61-2.84)	1.21 (0.70-2.07)	0.42 (0.09-1.95)
**Symptoms of OH**	1	2.34 (0.87-6.27)	0.70 (0.27-1.79)	NA[1]
**Blood pressure-lowering medications**				
Renin-angiotensin-aldosterone system inhibitors	1	0.69 (0.31-1.52)	0.97 (0.58-1.63)	0.96 (0.31-2.96)
Calcium channel blockers	1	0.49 (0.16-1.50)	0.88 (0.47-1.66)	1.42 (0.42-4.84)
Beta-blockers	1	0.66 (0.31-1.40)	1.19 (0.72-1.95)	1.89 (0.64-5.56)
Loop diuretics	1	0.86 (0.37-1.98)	0.62 (0.34-1.14)	0.81 (0.21-3.08)
Thiazide diuretics	1	0.98 (0.34-2.80)	1.40 (0.71-2.73)	1.63 (0.42-6.36)
Spironolactone	1	NA[1]	0.86 (0.30-2.47)	NA[1]
**Other medications**				
Nitrates[2]	1	1.18 (0.41-3.42)	2.52 (1.30-4.88)	2.14 (0.54-8.47)
Statins	1	1.65 (0.74-3.69)	1.72 (0.98-3.03)	1.41 (0.42-4.78)
Antidepressants	1	1.94 (0.73-5.17)	0.99 (0.44-2.23)	NA[1]
Sedatives (incl. sleeping medication)	1	0.90 (0.36-2.24)	0.85 (0.45-1.61)	0.34 (0.04-2.76)
**Polypharmacy (≥5 medications)**	1	0.77 (0.38-1.58)	1.22 (0.75-2.01)	1.51 (0.50-4.52)
**Assessments of fall risk**				
Downton Fall Risk Index ≥3 points[3]	1	1.05 (0.42-2.64)	1.42 (0.73-2.74)	1.12 (0.29-4.29)
Timed Up and Go ≥13.5 seconds[3]	1	1.12 (0.52-2.41)	0.89 (0.53-1.50)	0.97 (0.31-3.03)
**Assessments of functional ability**				
EQ-5D (no difficulties)	1	0.71 (0.34-1.49)	0.75 (0.46-1.25)	0.43 (0.14-1.30)
PADL (fully independent)	1	0.64 (0.25-1.65)	0.86 (0.42-1.76)	1.93 (0.24-15.62)
IAM (fully independent)	1	0.43 (0.14-1.34)	0.72 (0.34-1.52)	0.68 (0.15-3.13)
	**Mean (95% CI)**	**Mean (95% CI)**	**Mean (95% CI)**	**Mean (95% CI)**
**Supine blood pressure (mmHg)**				
Systolic	147.4 (144.0-150.7)	148.4 (141.7-155.2)	155.8 (151.9-159.7)	161.9 (151.1-172.7)
Diastolic	71.5 (69.9-73.1)	73.0 (69.7-76.2)	76.1 (74.2-78.0)	80.8 (75.6-86.0)
**Health care contacts**				

Primary care (visits)	8.0 (5.7-10.3)	8.8 (4.2-13.4)	10.4 (7.7-13.1)	8.5 (1.1-15.8)
Emergency department (visits)	0.5 (0.3 to 0.6)	0.4 (0.1 to 0.7)	0.5 (0.3 to 0.6)	0.4 (-0.1 to 0.8)
Inpatient care (nights)	1.6 (0.8 to 2.4)	1.0 (-0.6 to 2.7)	1.5 (0.5 to 2.4)	0.9 (-1.7 to 3.5)
**Assessments of fall risk**				
Downton Fall Risk Index (points)[3]	3.6 (3.4-3.9)	3.8 (3.3-4.2)	3.7 (3.4-4.0)	3.4 (2.6-4.1)
Timed Up and Go (seconds)[3]	16.7 (15.6-17.7)	18.4 (16.4-20.5)	16.5 (15.3-17.7)	15.3 (11.9-18.7)
**Assessments of cognitive functions**				
Mini-Mental State Examination (points)	27.4 (26.9-27.9)	26.5 (25.5-27.5)	27.3 (26.7-27.9)	27.0 (25.4-28.6)
NYU-PRT, immediate (points)	5.3 (4.8-5.7)	5.1 (4.2-6.0)	5.4 (4.9-5.9)	5.2 (3.8-6.6)
NYU-PRT, delayed (points)	5.5 (4.9-6.0)	5.2 (4.2-6.3)	5.6 (5.0-6.2)	4.7 (3.1-6.3)
30-item Naming Test (points)	22.5 (21.7-23.3)	23.6 (22.0-25.3)	23.8 (22.8-24.7)	22.9 (20.3-25.5)
Symbol Digit Modalities Test (points)	21.5 (20.1-22.9)	22.8 (19.9-25.7)	22.5 (20.8-24.2)	19.5 (15.1-23.9)
CLOX and Cube Copying Test (points)	10.2 (9.8-10.5)	9.3 (8.6-10.0)	9.7 (9.3-10.1)	10.0 (8.9-11.1)
Token Test short version (points)	4.6 (4.4-4.8)	4.3 (3.8-4.8)	4.5 (4.2-4.7)	3.8 (3.1-4.6)
The PaSMO (seconds)[3]	116.4 (107.9-124.9)	111.7 (93.1-130.2)	103.2 (93.3-113.1)	103.6 (71.4-135.9)
The Rey-Complex Figure test (points)	16.5 (16.0-16.9)	16.0 (15.1-16.9)	16.2 (15.7-16.8)	16.1 (14.7-17.5)
Victoria Stroop Test 1 (seconds)[3]	23.1 (20.7-25.4)	22.6 (17.9-27.3)	22.1 (19.3-24.9)	20.7 (13.3-28.2)
Victoria Stroop Test 2 (seconds)[3]	34.1 (31.4-36.8)	36.9 (31.5-42.4)	35.8 (32.6-39.0)	31.3 (22.9-39.8)
Victoria Stroop Test 3 (seconds)[3]	50.8 (46.4-55.2)	57.5 (48.9-66.2)	52.8 (47.7-57.9)	60.4 (46.9-73.9)
Trail Making Test A (seconds)[3]	76.8 (70.3-83.3)	72.5 (59.6-85.5)	75.6 (68.0-83.2)	84.7 (64.8-104.5)

1 No occurrences.

2 Nitrates includes both short-acting sublingual preparations and long-acting oral preparations.

3 Lower score implies better cognitive function. For the other tests, higher score implies better cognitive function.

CLOX, The Clock Drawing Task; IAM, Instrumental Activity Measure; NYU-PRT, New York University Paragraph Recall Test; OH, orthostatic hypotension; PADL, Personal Activities of Daily Living; PaSMO, Parallel Serial Mental Operations.

Supplementary Table 3. The odds ratios, means and 95% CI for the baseline results in relation to orthostatic hypotension status after multiple adjustments (model 3).

	**No OH**, n = 158	**Classical OH**		**Delayed OH**, n = 15
		**Transient**, n = 38	**Continuous**, n = 113	
	**OR (95% CI)**	**OR (95% CI)**	**OR (95% CI)**	**OR (95% CI)**
**Living in a nursing home**	1	NA[1]	0.87 (0.06-11.85)	NA[1]
**Neurological disease or dementia**	1	0.93 (0.31-2.78)	0.59 (0.27-1.25)	NA[1]
**Atrial fibrillation on electrocardiogram**	1	0.24 (0.05-1.21)	0.86 (0.38-1.92)	1.23 (0.27-5.69)
**Elevated supine blood pressure**	1	0.79 (0.34-1.83)	2.21 (1.16-4.21)	8.86 (1.01-77.70)
**Abnormal pulse response**	1	1.37 (0.61-3.09)	1.53 (0.86-2.73)	0.45 (0.09-2.21)
**Symptoms of OH**	1	2.77 (0.86-8.91)	1.05 (0.36-3.07)	NA[1]
**Blood pressure-lowering medications**				
Renin-angiotensin-aldosterone system inhibitors	1	1.19 (0.43-3.25)	1.13 (0.58-2.21)	0.54 (0.12-2.48)
Calcium channel blockers	1	0.46 (0.12-1.73)	0.89 (0.43-1.85)	0.92 (0.22-3.91)
Beta-blockers	1	0.99 (0.39-2.48)	1.12 (0.60-2.10)	1.93 (0.50-7.45)
Loop diuretics	1	1.14 (0.34-3.77)	0.77 (0.34-1.72)	0.58 (0.10-3.39)
Thiazide diuretics	1	1.07 (0.34-3.34)	1.34 (0.63-2.82)	2.00 (0.41-9.77)
Spironolactone	1	NA[1]	1.17 (0.36-3.75)	NA[1]
**Other medications**				
Nitrates[2]	1	1.11 (0.31-3.95)	3.38 (1.53-7.50)	2.61 (0.45-15.22)
Statins	1	2.66 (0.95-7.47)	1.61 (0.81-3.20)	1.29 (0.30-5.58)
Antidepressants	1	1.68 (0.55-5.11)	0.79 (0.31-2.02)	NA[1]
Sedatives (incl. sleeping medication)	1	0.92 (0.33-2.52)	0.57 (0.27-1.21)	0.37 (0.04-3.26)
**Polypharmacy (≥5 medications)**	1	1.08 (0.40-2.90)	1.87 (0.95-3.67)	2.77 (0.61-12.52)
**Assessments of fall risk**				
Downton Fall Risk Index ≥3 points[3]	1	1.40 (0.50-3.92)	1.83 (0.85-3.93)	1.31 (0.26-6.50)
Timed Up and Go ≥13.5 seconds[3]	1	1.02 (0.44-2.34)	0.90 (0.50-1.62)	1.08 (0.29-3.97)
**Assessments of functional ability**				
EQ-5D (no difficulties)	1	0.68 (0.30-1.56)	0.70 (0.39-1.26)	0.43 (0.11-1.59)
PADL (fully independent)	1	0.76 (0.23-2.45)	0.65 (0.28-1.50)	1.49 (0.13-16.82)
IAM (fully independent)	1	0.32 (0.09-1.15)	0.69 (0.28-1.67)	1.15 (0.13-10.12)
	**Mean (95% CI)**	**Mean (95% CI)**	**Mean (95% CI)**	**Mean (95% CI)**
**Supine blood pressure (mmHg)**				
Systolic	139.6 (127.0-152.3)	139.9 (125.6-154.1)	147.0 (133.9-160.1)	154.2 (138.0-170.4)
Diastolic	74.3 (68.0-80.5)	76.2 (69.1-83.2)	78.8 (72.3-85.3)	83.4 (75.3-91.4)
**Health care contacts**				
Primary care (visits)	17.6 (8.6-26.6)	19.5 (9.4-29.6)	20.3 (11.0-29.6)	17.7 (6.2-29.3)

Emergency department (visits)	0.4 (-0.1 to 0.9)	0.4 (-0.1 to 1.0)	0.5 (-0.0 to 1.0)	0.4 (-0.3 to 1.0)
Inpatient care (nights)	1.1 (-2.2 to 4.3)	0.8 (-2.8 to 4.4)	1.5 (-1.8 to 4.8)	0.8 (-3.3 to 4.9)
**Assessments of fall risk**				
Downton Fall Risk Index (points)[3]	5.0 (4.3-5.7)	5.2 (4.4-6.0)	5.1 (4.4-5.9)	4.9 (4.0-5.8)
Timed Up and Go (seconds)[3]	19.0 (15.1-22.8)	20.1 (15.8-24.5)	18.8 (14.8-22.8)	17.6 (12.6-22.6)
**Assessments of cognitive functions**				
Mini-Mental State Examination (points)	23.0 (21.2-24.9)	22.1 (20.1-24.2)	22.6 (20.7-24.5)	22.2 (19.8-24.6)
NYU-PRT, immediate (points)	5.0 (2.9-7.2)	5.1 (2.8-7.4)	5.1 (3.0-7.3)	5.1 (2.6-7.6)
NYU-PRT, delayed (points)	4.6 (1.4-7.9)	4.7 (1.3-8.2)	4.6 (1.3-8.0)	3.9 (0.2-7.6)
30-item Naming Test (points)	20.3 (17.1-23.4)	21.5 (17.9-25.0)	21.6 (18.3-24.9)	20.7 (16.7-24.7)
Symbol Digit Modalities Test (points)	15.4 (9.1-21.7)	17.5 (10.6-24.4)	16.4 (9.9-22.8)	13.8 (6.2-21.4)
CLOX and Cube Copying Test (points)	8.8 (7.5-10.2)	8.3 (6.8-9.8)	8.3 (6.9-9.7)	8.5 (6.8-10.2)
Token Test short version (points)	4.0 (2.9-5.1)	3.8 (2.6-5.0)	3.9 (2.8-5.0)	3.2 (1.9-4.5)
The PaSMO (seconds)[3]	139.0 (89.5-188.5)	133.6 (80.6-186.6)	124.1 (72.4-175.9)	121.6 (61.1-182.1)
The Rey-Complex Figure test (points)	15.9 (13.8-17.9)	15.8 (13.6-18.0)	15.5 (13.4-17.5)	15.1 (12.7-17.6)
Victoria Stroop Test 1 (seconds)[3]	28.8 (17.5-40.2)	27.7 (15.3-40.1)	28.1 (16.5-39.6)	27.5 (13.8-41.1)
Victoria Stroop Test 2 (seconds)[3]	54.3 (42.6-66.0)	54.6 (41.9-67.3)	57.5 (45.6-69.4)	53.3 (39.3-67.4)
Victoria Stroop Test 3 (seconds)[3]	74.3 (54.1-94.5)	81.3 (59.3-103.3)	77.0 (56.5-97.6)	84.8 (60.6-109.1)
Trail Making Test A (seconds) [3]	84.8 (56.5-113.1)	79.0 (48.1-109.8)	81.6 (52.7-110.4)	90.5 (56.5-124.5)

1 No occurrences.

2 Nitrates includes both short-acting sublingual preparations and long-acting oral preparations.

3 Lower score implies better cognitive function. For the other tests, higher score implies better cognitive function.

CLOX, The Clock Drawing Task; IAM, Instrumental Activity Measure; NYU-PRT, New York University Paragraph Recall Test; OH, orthostatic hypotension; PADL, Personal Activities of Daily Living; PaSMO, Parallel Serial Mental Operations.

SUPPLEMENTARY DATA

© 2024. Geijerstam PAF et al. Published online at http://www.aginganddisease.org/EN/10.14336/AD.2024.0205
